# Expression Profiles and Biomarker Potential of Long Non-Coding RNAs H19, NEAT1, MALAT1 and HOTAIR in Locally Advanced Rectal Cancer Patients

**DOI:** 10.3390/ijms27041672

**Published:** 2026-02-09

**Authors:** Katarina Eric, Jovana Rosic Stojkovic, Marko Miladinov, Sandra Dragicevic, Goran Barisic, Velimir Markovic, Katarina Zeljic

**Affiliations:** 1 Department of Pathology, Pathohistology and Medical Cytology, University Clinical Center of Serbia, 11000 Belgrade, Serbia; ketrineric@gmail.com; 2 Faculty of Medicine, University of Belgrade, 11000 Belgrade, Serbia; jovana.rosic@med.bg.ac.rs (J.R.S.); barisic_goran@yahoo.com (G.B.); mbecambeca@yahoo.com (V.M.); 3 Clinic for Digestive Surgery–First Surgical Clinic, University Clinical Center of Serbia, 11000 Belgrade, Serbia; marko.miladinov90@gmail.com; 4 Gene Regulation in Cancer Group, Institute of Molecular Genetics and Genetic Engineering, University of Belgrade, 11000 Belgrade, Serbia; sandra.d@imgge.bg.ac.rs; 5 Faculty of Biology, University of Belgrade, 11000 Belgrade, Serbia

**Keywords:** rectal cancer, H19, NEAT1, MALAT1, HOTAIR, neoadjuvant chemoradiotherapy

## Abstract

Locally advanced rectal cancer (LARC) presents a clinical challenge due to lack of reliable molecular biomarkers for early diagnosis, prognosis, and prediction of response to neoadjuvant chemoradiotherapy (nCRT). Long non-coding RNAs (lncRNAs) have emerged as promising candidates due to their characteristics and regulatory roles. H19, NEAT1, MALAT1 and HOTAIR are lncRNAs deregulated in gastrointestinal cancers, with insufficient data on their biomarker potential in LARC. The study aimed to analyze the diagnostic, prognostic and predictive utility of H19, NEAT1, MALAT1 and HOTAIR in LARC. Relative expression was evaluated by RT-qPCR in tumor and non-tumor tissues from 25 LARC patients before and after nCRT. H19, NEAT1, and MALAT1 showed significantly altered expression in tumor tissue, as well as non-tumor tissue before and after nCRT. H19 expression was significantly higher in tumor vs. non-tumor tissue before treatment and demonstrated moderate potential to discriminate between tumor and non-tumor. None of the lncRNAs showed statistically significant predictive values for nCRT response or association with treatment outcomes in our study, which was limited by the small number of responders. Our results suggest that H19 might be considered as a potential therapeutic target in LARC. Further studies with larger patient groups are required to confirm its diagnostic and prognostic utility.

## 1. Introduction

Colorectal cancer (CRC) is a malignant tumor that originates in the epithelial lining of the large intestine. It is the third-most common cancer worldwide and the third leading cause of cancer-related death [1]. The most common histological type of CRC, accounting for 90% of cases, is adenocarcinoma [2]. Rectal cancer (RC) constitutes about one-third of all CRC cases [3]. Locally advanced rectal cancer (LARC) remains a significant clinical challenge in terms of diagnosis, prognosis and prediction of response to administered therapy. The diagnosis of LARC is primarily based on a combination of clinical assessment and radiological imaging [4]. Despite advancements in therapeutic strategies for LARC patients, overall disease prognosis remains unfavorable. LARC patients typically undergo a standard treatment protocol that includes neoadjuvant chemoradiotherapy (nCRT) followed by surgical total mesorectal excision (TME) and adjuvant chemotherapy [4]. Patients achieving a clinical complete response (cCR) after nCRT may enter a watch-and-wait protocol avoiding radical surgery, in which cCR is considered an approximation of pathological complete response (pCR), which is observed in 15–25% of patients [5,6]. There are no validated molecular biomarkers that can be reliably used for early diagnosis of RC, for prognosis, or for predicting therapy response. Due to the limited utility of clinicopathological and radiological biomarkers for early diagnosis, prognosis, and distinguishing responders from non-responders to nCRT, research has focused on molecular biomarkers, particularly non-coding RNAs. Among these, long non-coding RNAs (lncRNAs), RNA molecules longer than 200 nucleotides, have attracted significant scientific interest due to their roles in processes such as growth, proliferation, differentiation, apoptosis, etc. [7]. Additionally, lncRNAs interact with DNA, proteins, and other RNA molecules, including other non-coding RNAs, forming complex regulatory networks that influence biological processes and gene expression [8]. The role and significance of lncRNAs in the response to nCRT in LARC patients remains insufficiently elucidated, as does their importance for disease diagnosis and prognosis.

In this study, we focused on four lncRNAs selected based on existing evidence of their functional relevance in cancer biology and their potential as molecular cancer biomarkers. *H19* (11p15.5) is an imprinted, maternally expressed gene that is often deregulated in various cancer types, including CRC [9]. H19 lncRNA has been shown to impair the efficacy of chemo and radiation therapy in cancer, including CRC [10]. The *NEAT1* (Nuclear Paraspeckle Assembly Transcript 1) gene (11q13.1) encodes a long non-coding RNA that is deregulated in carcinomas of the gastrointestinal tract [11]. There is evidence that NEAT1 confers chemotherapy resistance in various cancer types [12]. *MALAT1* (Metastasis-Associated Lung Adenocarcinoma Transcript 1), 11q13.1, encodes lncRNA MALAT1, acting as a transcriptional regulator of genes involved in cell migration and cancer metastasis. MALAT1 has been recognized as an important player in tumorigenesis, as it regulates cancer-related pathways, including MAPK/ERK, PI3K/AKT, β-catenin/Wnt, and Hippo [13]. Data indicate that MALAT1 has the potential to serve as both a diagnostic and a predictive biomarker in CRC [14]. The HOX Transcript Antisense Intergenic RNA (HOTAIR), 12q13.13, is a long non-coding trans-acting RNA molecule that is frequently deregulated in cancers of the digestive tract and plays a role in chemoresistance [15]. H19, HOTAIR, and MALAT1 have been reported to be upregulated in colon cancer and are thus considered oncogenes [8]. Given the well-established roles of H19, NEAT1, MALAT1, and HOTAIR in CRC and the limited knowledge of their roles in LARC, a comprehensive investigation of their involvement in LARC is warranted and necessary.

The aim of this study was to explore the expression patterns of the lncRNAs H19, NEAT1, MALAT1, and HOTAIR and to assess their potential as biomarkers for diagnosis, disease prognosis, and prediction of nCRT treatment response in LARC patients. In addition, data from publicly available databases were analyzed to compare them with the findings from our LARC cohort. We hypothesize that the expression profiles of H19, NEAT1, MALAT1, and HOTAIR are altered following nCRT in LARC patients, and that these lncRNAs may be considered as potential diagnostic, prognostic, and predictive molecular biomarkers in LARC.

## 2. Results

### 2.1. Expression Profile of H19, NEAT1, MALAT1, and HOTAIR in LARC and Its Association with Demographic and Clinicopathological Characteristics of LARC Patients

**Figure 1:**
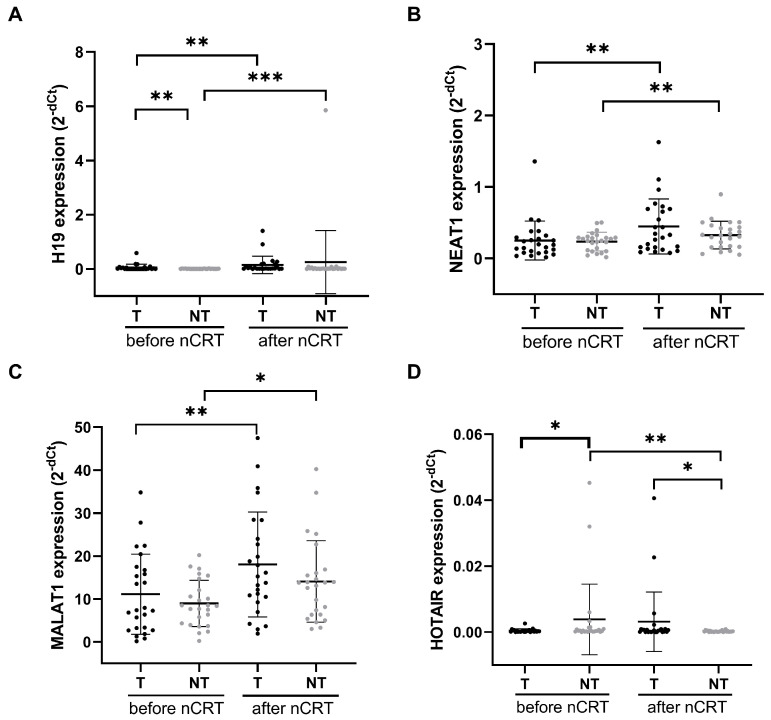
Relative expression levels of lncRNAs H19 (**A**), NEAT1 (**B**), MALAT1 (**C**) and HOTAIR (**D**) in tumor and adjacent non-tumor tissue of patients with locally advanced rectal carcinoma, measured before and after neoadjuvant chemoradiotherapy (nCRT). Data are presented as mean ± standard deviation (SD). * *p* < 0.05 ** *p* < 0.01 *** *p* < 0.001. T—tumor (*n* = 25), NT—non-tumor (*n* = 25).

Figure 1 shows the relative expression of the analyzed lncRNAs in tumor and non-tumor tissues from LARC patients before and after nCRT. The relative expression of H19, NEAT1 and MALAT1 in tumor tissue before nCRT was significantly lower than that in tumor tissue after nCRT (*p* = 0.002, *p* = 0.002, and *p* = 0.005, respectively). A similar pattern was observed in non-tumor tissue, where H19, NEAT1, and MALAT1 expression also differed significantly before and after nCRT (*p* = 0.0005, *p* = 0.004, and *p* = 0.012, respectively). For HOTAIR, no significant differences in expression were observed in the analyzed tumor tissues before and after nCRT (*p* = 0.070), but there was a difference in non-tumor tissues before and after nCRT (*p* = 0.009). H19 expression was significantly higher in tumor tissue compared with non-tumor tissue before nCRT (*p* = 0.001). In contrast, no significant differences in NEAT1 or MALAT1 expression were found between tumor and non-tumor tissue either before (*p* = 0.771 and *p* = 0.412, respectively) or after nCRT (*p* = 0.312 and *p* = 0.252, respectively). For HOTAIR, there was significant difference in expression between tumor and non-tumor tissues before nCRT (*p* = 0.029), and expression was significantly higher in tumor tissues than in non-tumor tissues after nCRT (*p* = 0.034).

The association between the relative expression of H19, NEAT1, MALAT1, and HOTAIR in rectal tumor tissue before and after nCRT, and the demographic and clinicopathological characteristics of LARC patients, is shown in Supplementary Materials Tables S1 and S2. No associations between clinicopathological characteristics and the expression of the analyzed lncRNAs were observed.

### 2.2. Diagnostic, Prognostic and Predictive Potential of H19, NEAT1, MALAT1, and HOTAIR Expression in LARC

**Figure 2:**
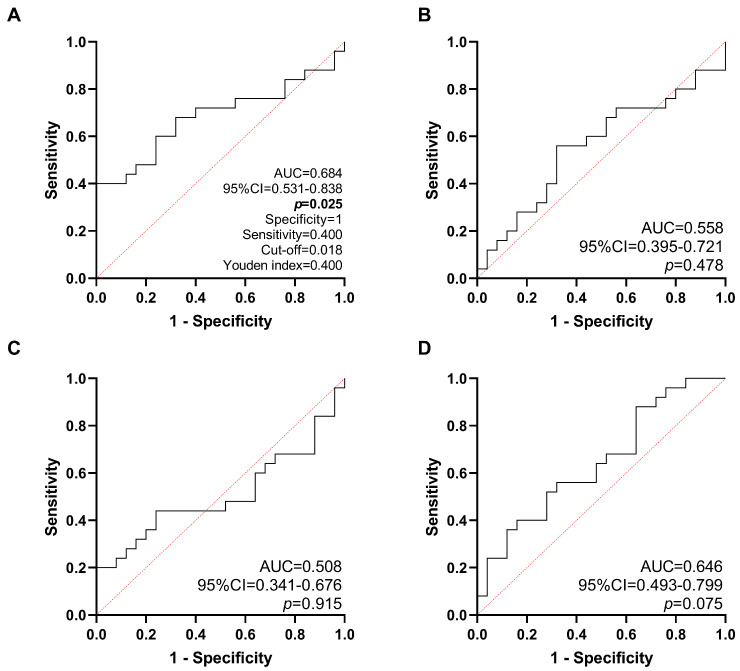
Diagnostic potential of the expression of H19 (**A**), NEAT1 (**B**), MALAT1 (**C**) and HOTAIR (**D**) before neoadjuvant chemoradiotherapy (nCRT) to distinguish tumor from non-tumor tissue. Black solid lines indicate ROC curves; red dotted lines indicate the reference lines; AUC—area under curve; 95% CI—95% confidence interval.

The diagnostic potential of H19, NEAT1, MALAT1, and HOTAIR expression to discriminate between tumor and non-tumor tissue was evaluated by ROC curve analysis. H19 expression measured before nCRT has moderate, but promising potential to discriminate between tumor and non-tumor (AUC = 0.684, 95% CI = 0.531–0.838, *p* = 0.025) (Figure 2). In contrast, NEAT1 expression did not help distinguish tumor from healthy tissue (AUC = 0.558, 95% CI = 0.395–0.721, *p* = 0.478). Similarly, MALAT1 expression showed no diagnostic value, with an AUC of 0.508 (95% CI = 0.341–0.676, *p* = 0.915). The same trend was found for HOTAIR (AUC = 0.646, 95% CI = 0.493–0.799, *p* = 0.075) (Figure 2).

**Figure 3:**
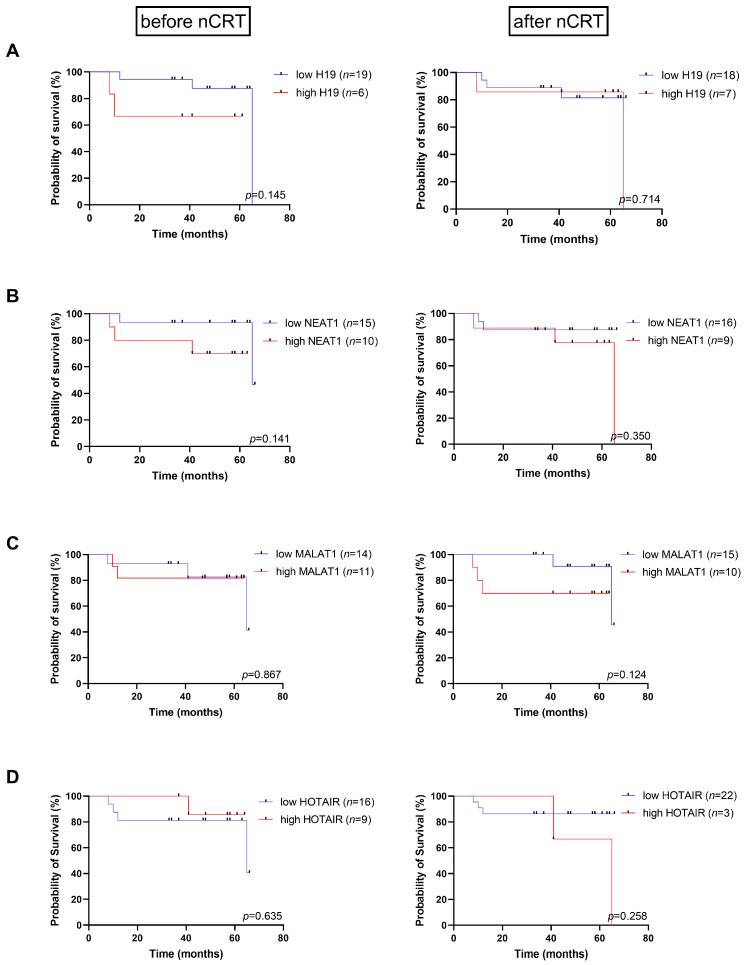
Kaplan–Meier curves of overall survival in patients with locally advanced rectal cancer depending on H19 (**A**), NEAT1 (**B**), MALAT1 (**C**), and HOTAIR (**D**) expression levels before and after neoadjuvant chemoradiotherapy (nCRT). Low and high levels of relative expression refer to values above or below the median.

To determine the potential of H19, NEAT1, MALAT1, and HOTAIR as prognostic biomarkers, lncRNA expression levels were categorized as low or high based on the median (Supplementary Materials Table S3). Kaplan–Meier survival curve analysis revealed no differences in overall survival between LARC patients with low and high expression of H19, NEAT1, MALAT and HOTAIR in tumor tissue before nCRT (*p* = 0.145, *p* = 0.141, *p* = 0.867 and *p* = 0.635, respectively) and after nCRT (*p* = 0.714, *p* = 0.350, *p* = 0.125 and *p* = 0.258, respectively) (Figure 3). There were no significant differences in disease-free survival by H19, NEAT1, MALAT1, and HOTAIR expression in the tumor before and after nCRT (Supplementary Materials Figure S1). According to Cox regression hazard risk analysis, none of the analyzed lncRNAs can be used as predictors of death or disease recurrence (Supplementary Materials Table S4).

Among the 25 LARC patients in our study group, only 3 (12%) were classified as responders to nCRT based on the Mandrad tumor regression grading system (Table 1). Relative expression of H19, NEAT1, MALAT1, and HOTAIR in tumor tissue before nCRT did not show differences between responders and non-responders, suggesting it cannot be used to predict pathological response, as determined by TRG (*p* = 0.558 *p* = 0.503, *p* = 0.676, *p* = 0.738, respectively).

### 2.3. Analysis of Public Databases and Web-Based Platforms: Expression Profile and Biomarker Potential of H19, NEAT1, MALAT1, and HOTAIR

**Figure 4:**
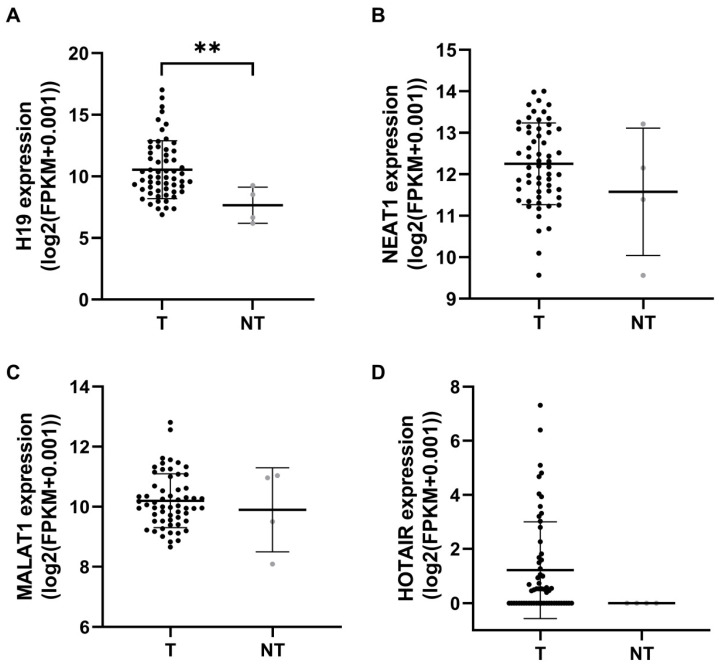
Expression levels of lncRNAs H19 (**A**), NEAT1 (**B**), MALAT1 (**C**), and HOTAIR (**D**) in tumor and non-tumor tissue of patients with rectal cancer, retrieved from the UCSC Xena Browser (GDC TCGA READ data). Data are presented as mean ± standard deviation (SD). ** *p* < 0.01. T—tumor (*n* = 59); NT—non-tumor (*n* = 4).

We utilized the GDC TCGA READ project by UCSC Xena to assess lncRNA expression profiles in rectal cancer (II and III stages) compared to normal tissue. The expression levels of H19 were significantly higher in rectal tumor tissue compared to normal tissue (*p* = 0.009), whereas no significant differences were observed for NEAT1, MALAT1 and HOTAIR (*p* = 0.402, p = 0.692, *p* = 0.074, respectively) (Figure 4). Due to the small number of matched samples (only two pairs), statistical analysis was not performed for the matched tissue samples from the GDC TCGA READ project. We also utilized the TNM plot platform to analyze lncRNA expression profiles in rectal cancer versus normal tissues (Supplementary Materials Figure S2). TNM plot analysis revealed that H19, NEAT1, and MALAT1 were significantly downregulated in rectal adenocarcinoma compared to normal tissue, when unpaired samples were analyzed (*p* = 8.27 × 10^−32^, *p* = 4.47 × 10^−55^, *p* = 3.74 × 10^−44^, respectively). In contrast, H19 and HOTAIR showed higher expression levels in rectal cancer tissues when paired rectal adenocarcinoma and adjacent normal tissue were analyzed (*p* = 3.43 × 10^−1^, *p* = 3.55 × 10^−2^, respectively). In the GEPIA 2 interactive web server, which contains RNA sequencing data from TCGA and GTEx projects, for READ, NEAT1 was significantly downexpressed in tumor compared to normal tissue (adj *p* = 9.67 × 10^−38^), as was MALAT1 (adj *p* = 1.51 × 10^−42^) (Supplementary Materials Figure S3).

To estimate the prognostic potential of the lncRNAs analyzed in our study, we used the KMplot web platform (Supplementary Materials Figure S4). A significant difference in survival curves was observed among rectal adenocarcinoma patients based on H19 and NEAT1 expression. Patients with high H19 expression had poorer overall survival compared to those with low expression (*p* = 0.01, log-rank test), while NEAT1 low-expressing patients had shorter survival (*p* = 0.0081, log-rank test). In contrast, no significant differences in survival were observed for MALAT1 and HOTAIR (Supplementary Materials Figure S4).

**Figure 5:**
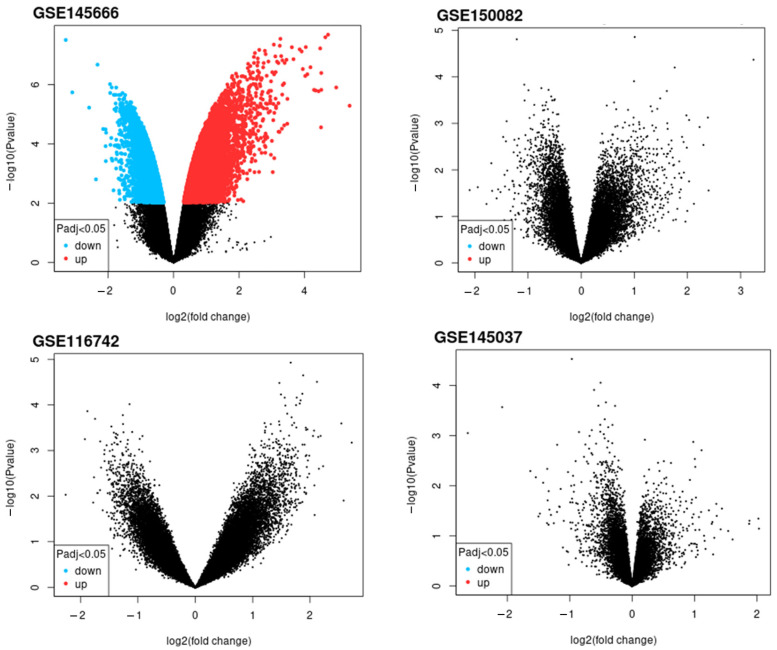
Volcano plots of differentially expressed genes between responders and non-responders from the analyzed GEO datasets. Blue dots represent significantly downregulated genes, red dots represent significantly upregulated genes, while black dots are non-significant genes. An adjusted *p* value < 0.05 was considered significant. vs.—versus.

The potential of the lncRNAs analyzed in this study as predictive biomarkers was assessed in an independent cohort from the public GEO database. In GSE150082, GSE116742 and GSE145037 datasets, neither of our target lncRNAs was significantly differentially expressed between LARC patients with good and poor response, whereas in GSE145666, NEAT1 was identified among the top significantly deregulated genes (Figure 5). According to the ROC Plotter web platform, NEAT1, MALAT1, and HOTAIR did not show predictive potential for response to 5-FU and radiotherapy, which is consistent with our results (Supplementary Materials Figure S5).

## 3. Discussion

Since LARC is a life-threatening malignancy, further molecular characterization is essential to improve treatment strategies and patient outcomes. It is crucial to identify and characterize molecular signatures of reliable biomarkers for the diagnosis, prognosis, and prediction of LARC. Among non-coding RNAs, lncRNAs are a class that remains poorly understood and understudied. lncRNAs are stable in tissue and have emerged as promising therapeutic targets in various cancers, including rectal cancer. There are a limited number of studies in the literature investigating the potential of lncRNAs as biomarkers in LARC. Although various lncRNAs have been previously investigated, to our knowledge, H19, NEAT1, MALAT1, and HOTAIR have not been thoroughly analyzed in this context in LARC. We have analyzed lncRNA expression in biological samples and utilized multiple platforms and datasets to cross-check our findings on the diagnostic, prognostic, and predictive significance of H19, NEAT1, MALAT1, and HOTAIR in LARC patients.

Based on our results regarding H19 expression in tumor and non-tumor tissue before nCRT, H19 might be considered as an oncogenic lncRNA, as its expression is higher in tumor tissue, as confirmed by analysis of GDC TCGA data, GEPIA2, and TNM plot when matched tumor and normal samples were analyzed. Relative H19 expression before nCRT may be a moderate diagnostic biomarker for sensitive discrimination of tumor from non-tumor tissue in LARC. The diagnostic potential of both tumor and serum H19 has been reported in gastric cancer [16,17], and CRC [18,19]. There were no differences in NEAT1 and MALAT1 expression between tumor and non-tumor tissue, either before or after nCRT. These results were consistent with those obtained from analysis of GDC TCGA-READ data. In contrast, in GEPIA2 and TNM plot, expression of both NEAT1 and MALAT1 was significantly lower in rectal tumors than in normal tissue. These discrepancies may, at least in part, be attributed to differences in the transcript isoforms detected by the assays used. Contrary to our results for NEAT1, serum NEAT1 was considered as diagnostic and prognostic biomarker in CRC [20], and a diagnostic biomarker in prostate cancer [21]. Numerous studies report increased MALAT1 in CRC, as previously reviewed in the literature [22].

To cross-validate our findings on differential expression of H19, NEAT1, MALAT1 and HOTAIR in rectal adenocarcinoma compared with normal tissue, we used multiple web-based platforms. Specifically, we analyzed GDC TCGA data via the UCSC Xena platform; GEPIA2, which integrates TCGA and GTEx data; and TNM plot, which includes transcriptomic data from TCGA, GTEx, TARGET, and GEO. The results may vary depending on the database and software used, and in some cases, they may even be contradictory. One possible reason for the observed discrepancies is that we specifically filtered for stage II and III rectal cancer in the TCGA database via UCSC Xena, whereas, to the best of our knowledge, such stage-specific filtering was not available on the GEPIA2 and TNM plot platform. In addition, different normalization methods and statistical analyses, as well as biological variability, such as intra- and inter-tumor heterogeneity, may contribute to conflicting results across different validation platforms. Therefore, conducting expression analyses in clinical samples remains essential, and confirmatory studies are crucial to validate bioinformatics findings.

The significant differences in H19, NEAT1, and MALAT1 expression in both tumor and non-tumor tissues before and after nCRT suggest that these lncRNAs respond to the therapy by increasing their expression. This response implies that H19, NEAT1, and MALAT1 may act as potential therapeutic targets, as they are somewhat intertwined in a complex regulatory network that is activated in cellular response to radiation and chemotherapy. Also, the increase in H19, NEAT1, and MALAT1 expression might reflect changes in tissue composition following nCRT, including inflammation or treatment-induced necrosis. In addition, an increase in expression upon nCRT might suggest that H19, NEAT1, and MALAT have a potential protective or stress-response role in LARC, which should be further investigated through functional analysis. However, the mechanism by which nCRT affects H19, NEAT1, and MALAT1 expression remains unclear. H19 has been shown to play an important role in chemo and radiotherapy resistance in cancers of the gastrointestinal tract [23]. However, its effects on therapy response appear dual and context-dependent, varying across tumor types [23]. This variability may be attributed to factors such as tumor heterogeneity, the specific chemotherapeutic agents used, differences in molecular tumor profiles and different signaling pathways activated by H19 [23]. High H19 expression has been reported to promote 5-FU resistance in CRC by sponging miR-194-5p and inducing autophagy through upregulation of SIRT1, a direct target of miR-194-5p [24]. It has been reported that NEAT1 promotes 5-FU resistance in CRC [25] and that NEAT1 knockdown in CRC cells enhances 5-FU sensitivity by targeting miR-34a [26]. There was an association between high MALAT1 expression and 5-FU resistance in early stages of colon cancer [27]. Further functional analyses are needed to elucidate the effects of chemotherapy alone or in combination with radiotherapy, as well as its effects on lncRNAs. Several potential therapeutic strategies have been proposed to target overexpressed lncRNAs in cancer, aiming to reduce their expression. There are transcriptional approaches, such as gene-editing techniques to inhibit transcription, and post-transcriptional approaches, including the use of antisense oligonucleotides, small interfering RNAs, or even steric inhibition of lncRNA–protein interactions [28]. These approaches might be explored in combination with nCRT to reduce lncRNA expression and potentially enhance nCRT efficacy. Given that H19, NEAT1, and MALAT1 are evolutionarily conserved, pre-clinical studies in mammalian models are feasible [28].

Regarding prognostic potential, this study was limited by the number of events (5 deaths, 8 recurrences); therefore, the absence of a statistically significant prognostic value of the investigated lncRNAs should be considered exploratory rather than confirmatory. However, a recent meta-analysis comprising eight studies found that high NEAT1 expression is a predictor of overall survival, tumor differentiation, and tumor size in rectal cancer [29]. High H19 expression was reported as a predictor of poor overall and disease-free survival in CRC patients [18]. The inconsistencies between our study and previous studies may be explained by differences in the number of deaths and recurrence events among studies, as well as by the follow-up period and the criteria used to classify lncRNA expression into low- and high-expression groups. We used the median expression level to dichotomise of lncRNA expression, as this is the most commonly used approach in the literature. The small number of respondents in our study implies that the results regarding the predictive value of the analyzed lncRNAs should be interpreted with caution due to decreased statistical power. It is important to expand the cohort of responders in future studies to assess the predictive potential of these lncRNAs accurately. Even with exploration analysis, our results were in line with the ROC Plotter analysis. However, we should be aware of a key limitation of using ROC Plotter-the lack of information on whether the administered therapy in rectal adenocarcinoma cases was neoadjuvant, and how the response to therapy was assessed. This may impact the interpretation of these results.

A notable limitation of our study is the small number of LARC patients included, which can be attributed to strict inclusion criteria and the logistical complexity of the study. Because tumor and non-tumor tissue samples were collected from each patient both prior to and several weeks after nCRT, some patients elected to continue treatment at another center, which affected the total number of LARC patients ultimately included in the study. This may have impacted the final number of LARC patients included. It is noteworthy that the number of deaths and recurrence events in our study group was small, which made the analysis of prognostic significance exploratory. In addition, one limitation is small number of LARC patients who were good responders (TRG1, TRG2), which, in our cohort, was at the lower end of previously reported response rates. When analyzing potential biomarker expression, it is recommended to include liquid biopsies (e.g., plasma or serum) alongside solid tumor tissue. It is worth mentioning that GAPDH was the only endogenous control used in this study. Given the observed trend toward GAPDH expression instability induced by the nCRT treatment, further studies would benefit from the inclusion of multiple endogenous controls, enabling the identification of the most stable or the use of a geometric mean of at least three validated endogenous controls. In addition, the observed upregulation of target lncRNAs should be interpreted with caution, as it may be partially influenced by the variability of the reference gene under nCRT conditions. Despite limitations, our results provide valuable insights into the comprehensive identification of the diagnostic, prognostic and predictive potential of H19, NEAT1, MALAT1, and HOTAIR in LARC and lay the groundwork for future studies.

To conclude, our data suggest that H19, NEAT1, and MALAT1 may potential therapeutic targets in LARC, while H19 should also be investigated for its diagnostic potential in LARC patients. Further studies with a larger group of LARC patients and a prolonged follow-up period are required to confirm prognostic utility of H19. In addition, future studies should incorporate comprehensive omics data alongside radiomic and other clinical data.

## 4. Material and Methods

### 4.1. Ethical Aspects and Study Group

The current study was performed in line with the principles of the Declaration of Helsinki and approved by the Ethics Committee of the Faculty of Medicine of the University of Belgrade (approval number 1550/V-2, 31 May 2019) and the Ethics Committee of the University Clinical Center of Serbia (approval number 1228/11, 3 July 2025). Written informed consent was obtained from all participants included in the study.

The study group consisted of 25 patients with LARC (T3/T4, N0, M0 or any T, N1/N2, M0), treated at the Clinic for digestive surgery—First Surgical Clinic, University Clinical Center of Serbia, Belgrade—during the period from April 2019 to October 2020. Inclusion criteria: subjects with LARC, histopathological confirmation of intestinal-type adenocarcinoma from pre-treatment biopsies, and patients who underwent a long-course radiation treatment followed by surgical intervention (TME). Exclusion criteria: stage I or IV of the disease at presentation, and histopathologically verified mucinous adenocarcinoma of the rectum in initial biopsies.

Clinical tumor-node-metastasis (cTNM) staging was determined by magnetic resonance imaging (MRI), which confirmed the presence of LARC. All individuals included in the study underwent nCRT, which involved a total radiation dose of 50.4 Gy, administered in 28 fractions, in combination with two cycles of chemotherapy. The chemotherapy consisted of 5-fluorouracil (5-FU) at a dose of 425 mg/m^2^ and leucovorin at 20 mg/m^2^, administered in the first and fifth week of radiotherapy. This was followed by a surgical procedure, TME, which was performed 8–12 weeks after nCRT.

Patients were followed up every 3 months during the first year after surgery, then every 6 months during the second year, and thereafter annually. The last annual follow-up was conducted in September 2024. Overall survival was defined as the time from LARC diagnosis to death from any cause. Disease-free survival was defined as the time from the surgery to the date of confirmed recurrence. Patients who died without evidence of recurrence were censored at that time.

### 4.2. Biological Samples, Clinical and Histopathological Analyses

Prior to treatment, both tumor and non-tumor (adjacent healthy mucosa) tissue samples were obtained by anoscopy or rectoscopy, depending on tumor location: lower rectum (within 5 cm of the anal verge) or mid-rectum (5–10 cm from the anal verge), respectively. Post-treatment tumor and non-tumor samples were taken from the surgical specimen immediately after the operation. Collected tissue samples were placed in TRIzol^TM^ Reagent Solution (Invitrogen, Carlsbad, CA, USA) and stored at −80 °C until further use.

Each collected tissue specimen (tumor and non-tumor tissue obtained from the same anatomical region) was submitted for routine histopathological examination by an experienced pathologist. Samples histopathologically confirmed to be free of malignant cells were classified as non-tumor tissue. Tumor biological specimens were confirmed histopathologically as intestinal type adenocarcinoma on tumor biopsies before nCRT. Pathological TNM (pTNM) staging was performed according to the 8th edition of the American Joint Committee on Cancer (AJCC) from 2017. The degree of regression of rectal cancer on nCRT was confirmed by analysis of surgical resection specimens. Tumor response to preoperative treatment was estimated using Mandard’s tumor regression grade (TRG) system. Patients were classified as responders (TRG1, TRG2) and non-responders (TRG3, TRG4, TRG5) based on TRG histopathological category from the postsurgical specimen.

All demographic and clinicopathological characteristics of LARC patients were collected from medical records and summarized in Table 1.

### 4.3. RNA Isolation, cDNA Synthesis and Relative Expression of lncRNA

From collected tissue samples (tumor and non-tumor before and after nCRT), total RNA was isolated using TRIzol^TM^ Reagent Solution (Invitrogen, USA) according to the manufacturer’s protocol. Total RNA concentration and purity were measured using Ultrospec 3300 Pro spectrophotometer (Amersham Biosciences, Buckinghamshire, UK).

cDNA was synthesized by the High-Capacity cDNA Reverse Transcription kit (Applied Biosystems™, Foster City, CA, USA). The reaction consisted of 20 ng of RNA, 10× RT buffer, 25× dNTP mix, 10× random primers, Multiscribe reverse transcriptase, and RNA inhibitor. cDNA synthesis was performed at the following temperatures: 25 °C for 10 min, 37 °C for 120 min and 85 °C for 5 min.

Relative expression of target lncRNA was measured by Real Time PCR and TaqMan^TM^ gene expression assay (Thermo Fisher Scientific, Waltham, MA, USA) targeting H19 (ID Hs00262142_g1), NEAT1 (ID Hs01008264_s1), MALAT1 (ID Hs00273907_s1), HOTAIR (ID Hs03296680_s1) and GAPDH (ID Hs99999905_m1). The temperature profile of the real-time PCR reaction was as follows: 10 min at 95 °C, followed by 40 cycles of 15 s at 95 °C and 1 min at 60 °C. Normalization was performed using the endogenous control GAPDH, which showed no significant change in expression levels in tumor samples before and after nCRT, as well as in non-tumor samples (Supplementary Materials Table S5). All reactions were performed in duplicate, and the average Ct value was calculated for every sample. Data were analyzed by the 2^−dCt^ method [30].

### 4.4. Analysis of Public Databases and Web-Based Platforms

The expression levels of H19, NEAT1, MALAT1, and HOTAIR were analyzed in normal tissue and rectal cancer using data from the Genomic Data Commons—The Cancer Genome Atlas (GDC-TCGA), specifically from the Rectal Adenocarcinoma (READ) project. The GDC-TCGA READ dataset was accessed and analyzed through the open online platform UCSC Xena (University of California Santa Cruz Xena; http://xena.ucsc.edu; accessed on 26 May 2025) [31]. The TCGA-GDC READ project comprises 187 samples. Filtering was performed based on tissue type, retaining only normal tissue and primary rectal adenocarcinoma samples, as well as expression data for the lncRNAs of interest and AJCC pathological stages II and III. None of the patients with rectal cancer had a history of nCRT. After filtering, 64 samples remained for analysis, including 4 normal tissue samples and 60 tumor tissue samples. Only 2 matched pairs of normal and tumor tissues were available. The data were downloaded for subsequent statistical analysis.

Analysis of lncRNAs expression in rectal adenocarcinoma and normal tissue was also performed by TNM plot (https://tnmplot.com/analysis; accessed on 26 May 2025) [32] and GEPIA2 (http://gepia2.cancer-pku.cn/#index; accessed on 26 May 2025) [33].

Kaplan–Meier plotter (https://kmplot.com/analysis/; accessed on 26 May 2025) was used to analyze the prognostic value of lncRNA of our interest, and RNAseq datasets were used [34,35]. The analysis in the KMplot was restricted to rectal adenocarcinoma patients with II and III stage disease, identified as white, to ensure comparability with our study group. After filtering, 51 patients remained in the analysis. Patients were divided into high- and low-expression groups based on the median lncRNA expression level. Kaplan–Meier survival curves were compared using the log-rank test, and hazard ratios with 95% confidence intervals were calculated.

Gene Expression Omnibus (GEO) database (https://www.ncbi.nlm.nih.gov/geo/; accessed on 15 May 2025) was used to investigate the expression of the candidate lncRNA in LARC patients in relation to their response to nCRT. A combination of keywords was used to search the database: LARC, lncRNA, and long non-coding RNA. Four datasets (GSE145666, GSE150082, GSE116742, and GSE145037) were identified and used for further analysis (Supplementary Materials Table S6). Data were analyzed by web-based GEO2R software (https://www.ncbi.nlm.nih.gov/geo/geo2r/; accessed on 15 May 2025) with default settings and significance level cut-off set to *p* < 0.05 and log2 fold change = 0. To visualize differential expression, volcano plots were generated by GEO2R.

To analyze predictive value of lncRNAs in rectal cancer in response to chemo and radiotherapy, the web tool ROC Plotter was used (https://rocplot.com/; accessed on 4 July 2025) with JetSet settings [36].

The images obtained from the analysis were downloaded in their original form from the web-based platforms used. Details on the web-based platforms used are provided in Supplementary Materials Table S7.

### 4.5. Statistical Analysis

GraphPad Prism v.9 and SPSS v.21 were used for statistical analysis and visualization of the obtained results. Descriptive statistics as well as nonparametric and parametric statistical tests were used to analyze the results. Data normality was assessed using the Shapiro–Wilk test. Differences in the level of relative expression between the analyzed tumor tissue before and after nCRT and the non-tumor tissue before and after nCRT were determined using the Wilcoxon signed rank test or Student’s *t*-test, depending on the distribution of the data. For unpaired cases, the *t*-test was used for normally distributed data, whereas the Mann–Whitney U-test was used for non-normally distributed data. The association between the relative expression levels of selected lncRNAs in tumor tissue from LARC patients before and after nCRT and the patients’ demographic and clinicopathological characteristics is investigated using the nonparametric Pearson χ2 test or Fishers’ exact test. The diagnostic significance of each analyzed lncRNA was assessed using the Receiver Operating Characteristics (ROCs) curve, with the Area Under the Curve (AUC) and 95% confidence interval (95% CI). The optimal cut-off value was determined by the maximal Youden index (sensitivity + specificity − 1). The association between the relative levels of selected lncRNAs and overall and disease-free survival was assessed using Kaplan–Meier curves and the log-rank test. Cox-regression was used to calculate the hazard ratio (HR) with 95% CI. If the *p*-values obtained were less than 0.05, the results were considered significant.

## Figures and Tables

**Table 1:** Patients’ demographic and clinicopathological characteristics.

Characteristics		*n*	%
Sex	male	15	60
	female	10	40
Age, years (mean ± SD)	64.32 ± 11.022 (min: 34, max: 83)		
Age, years (median)	≤68	15	60
	>68	10	40
Clinical stage of disease	IIIB	7	26.9
	IIIC	19	73.1
T stage in clinical TNM	T3	18	72
	T4	7	28
N stage in clinical TNM	N1	7	28
	N2	18	72
M stage in clinical TNM	M0	25	100
Tumor regression grade (TRG) pathological	TRG1	2	8
	TRG2	1	4
	TRG3	10	40
	TRG4	12	48
Response to nCRT based on Mandard	responders (TRG1 + TRG2)	3	12
	non-responders (TRG3 + TRG4)	22	88
Pathological stage of disease	0	2	8
	I	5	20
	II	8	32
	III	9	36
	IV	1	4
T stage in pathological TNM	T0-Tis	2	8
	T1	1	4
	T2	6	24
	T3	15	60
	T4	1	4
N stage in pathological TNM	N0	15	60
	N1	9	36
	N2	1	4
M stage in pathological TNM	M0	24	96
	M1	1	4
Lymphatic invasion	L0	13	52
	L1	10	40
	Lx	2	8
Vascular invasion	V0	14	56
	V1	9	36
	Vx	2	8
Perineural invasion	PN0	18	72
	PN1	6	24
	missing	1	4
Disease outcome	alive	20	80
	death	5	20
Relapse occurrence	no	17	68
	yes	8	32
CEA before nCRT (IU/mL, mean ± SD)	17.96 ± 47.328		
CEA after nCRT (IU/mL, mean ± SD)	4.72 ± 5.969		
CA 19-9 before nCRT (IU/mL, mean ± SD)	28.17 ± 74.784		
CA 19-9 after nCRT (IU/mL, mean ± SD)	11.60 ± 10.148		

SD—standard deviation; TNM—tumor-node-metastasis; nCRT—neoadjuvant chemoradiotherapy; CEA—Carcinoembryonic Antigen; CA 19-9—Carbohydrate Antigen 19-9.

## Data Availability

The data presented in this study are available upon request from the corresponding author.

## Supplementary Materials

The following supporting information can be downloaded at https://www.mdpi.com/article/10.3390/ijms27041672/s1 [37,38,39,40].
