# Supplementary material for: Expression Profiles and Biomarker Potential of Long Non-Coding RNAs H19, NEAT1, MALAT1 and HOTAIR in Locally Advanced Rectal Cancer Patients
Source: Int J Mol Sci. 2026 Feb 9;27(4):1672. doi: 10.3390/ijms27041672 (PMC12940164; doi:10.3390/ijms27041672)
Supplement: Supplementary file 1 [file ijms-27-01672-s001.zip › ijms-4062981-supplementary.pdf]

Supplement Table S1. Association between the relative expression of H19, NEAT1, MALAT1 and HOTAIR in rectal tumor tissue before nCRT with demographic and clinicopathological characteristics of LARC patients.

Demographic and clinicopathological data		Relative expression before nCRT											
		H19		*p*	NEAT1		*p*	MALAT1		*p*	HOTAIR		*p*
		low *n* (%)	high *n* (%)		low *n* (%)	high *n* (%)		low *n* (%)	high *n* (%)		low *n* (%)	high *n* (%)	
**Sex**	female	6 (31.6)	4 (66.7)	0.126	5 (33.3)	5 (50)	0.405	6 (42.9)	4 (36.4)	0.742	6 (42.9)	4 (36.4)	0.734
	male	13 (68.4)	2 (33.3)		10 (66.7)	5 (50)		8 (57.1)	7 (63.6)		10 (62.5)	5 (55.6)	
**Age (years, median)**	<68	11 (57.9)	4 (66.7)	0.702	7 (46.7)	8 (80)	0.096	8 (57.1)	7 (63.6)	0.742	9 (56.2)	6 (66.7)	0.610
	>68	8 (42.1)	2 (33.3)		8 (53.3)	2 (20)		6 (42.9)	4 (36.4)		7 (43.8)	3 (33.3)	
**Clinical T stage**	T3	13 (68.4)	5 (83.3)	0.478	10 (66.7)	8 (80)	0.467	11 (78.6)	7 (63.6)	0.409	12 (75)	6 (66.7)	0.656
	T4	6 (31.6)	1 (16.7)		5 (33.3)	2 (20)		3 (21.4)	4 (36.4)		4 (25)	3 (33.3)	
**Clinical N stage**	N1	4 (21.1)	3 (50)	0.169	3 (20)	4 (40)	0.275	4 (28.6)	3 (27.3)	0.943	4 (25)	3 (33.3)	0.656
	N2	15 (78.9)	3 (50)		12 (80)	6 (60)		10 (71.4)	8 (72.7)		12 (75)	6 (66.7)	
**Pathological T stage**	T1+T2	6 (31.6)	3 (50)	0.412	6 (40)	3 (30)	0.610	7 (50)	2 (18.2)	0.100	7 (73.8)	2 (22.2)	0.282
	T3+T4	13 (68.4)	3 (50)		9 (60)	7 (70)		7 (50)	9 (81.8)		9 (56.2)	7 (77.8)	
**Pathological N stage**	N0	10 (52.6)	5 (83.3)	0.181	9 (60)	6 (60)	1.000	8 (57.1)	7 (63.6)	0.742	8 (50)	7 (77.8)	0.174
	N1+N2	9 (47.4)	1 (16.7)		6 (40)	4 (40)		6 (42.9)	4 (36.4)		8 (50)	2 (22.2)	
**Pathological disease stage**	0+I+II	10 (52.6)	5 (83.3)	0.181	9 (60)	6 (60)	1.000	8 (57.1)	7 (63.6)	0.742	8 (50)	7 (77.8)	0.174
	III	9 (47.4)	1 (16.7)		6 (40)	4 (40)		6 (42.9)	4 (36.4)		8 (50)	2 (22.2)	
**Response to therapy (Mandrad)**	responders (TRG1+TRG2)	2 (10.5)	1 (16.7)	0.687	2 (13.3)	1 (10)	0.802	2 (14.3)	1 (9.1)	0.692	3 (18.8)	0 (0)	0.166
	non-responders (TRG3+TRG4)	17 (89.5)	5 (83.3)		13 (86.7)	9 (90)		12 (85.7)	10 (90.9)		13 (81.2)	9 (100)	

n – total number of LARC patients
nCRT – neoadjuvant chemoradiotherapy

TRG – tumor regression grade

p<0.05 is shown in bold

Low and high level of relative expression refer to higher or lower that median (H19: median – 0.004, NEAT1: median - 0.190, MALAT: median — 7.383, HOTAIR: median – 0.0002). Median values are reported as 2^{-dCt}.

Supplement Table S2. Association between the relative expression of H19, NEAT1, MALAT1, and HOTAIR in rectal tumor tissue after nCRT with demographic and clinicopathological characteristics of LARC patients.

**Demographic and clinicopathological data**		**Relative expression after nCRT**											
		**H19**			**NEAT1**			**MALAT1**			**HOTAIR**		
		**low** *n* (%)	**high** *n* (%)	***p***	**low** *n* (%)	**high** *n* (%)	***p***	**low** *n* (%)	**high** *n* (%)	***p***	**low** *n* (%)	**high** *n* (%)	***p***
**Sex**	female	5 (27.8)	5 (71.4)	**0.045**	5 (31.2)	5 (55.6)	0.234	6 (40)	4 (40)	1.000	8 (36.4)	2 (66.7)	0.315
	male	13 (72.2)	2 (28.6)		11 (68.8)	4 (44.4)		9 (60)	6 (60)		14 (63.6)	1 (33.3)	
**Age (years, median)**	<68	11 (61.1)	4 (57.1)	0.856	8 (50)	7 (77.8)	0.174	7 (46.7)	8 (80)	0.096	14 (63.6)	1 (33.3)	0.315
	>68	7 (38.9)	3 (42.9)		8 (50)	2 (22.2)		8 (53.3)	2 (20)		8 (36.4)	2 (66.7)	
**Clinical T stage**	T3	13 (72.2)	5 (71.4)	0.968	12 (75)	6 (66.7)	0.656	12 (80)	6 (60)	0.275	16 (72.7)	2 (66.7)	0.826
	T4	5 (27.8)	2 (28.6)		4 (25)	3 (33.3)		3 (20)	4 (40)		6 (27.3)	1 (33.3)	
**Clinical N stage**	N1	5 (27.8)	2 (28.6)	0.968	6 (37.5)	1 (11.1)	0.158	5 (33.3)	2 (20)	0.467	7 (31.8)	0 (0)	0.250
	N2	13 (72.2)	5 (71.4)		10 (62.5)	8 (88.9)		10 (66.7)	8 (80)		15 (68.2)	3 (100)	
**Pathological T stage**	T1+T2	6 (33.3)	3 (42.9)	0.656	6 (37.5)	3 (33.3)	0.835	7 (46.7)	2 (20)	0.174	8 (36.4)	1 (33.3)	0.918
	T3+T4	12 (66.7)	4 (57.1)		10 (62.5)	6 (66.7)		8 (53.3)	8 (80)		14 (63.6)	2 (66.7)	
**Pathological N stage**	N0	9 (50)	6 (85.7)	0.102	9 (56.2)	6 (66.7)	0.610	10 (66.7)	5 (50)	0.405	13 (59.1)	2 (66.7)	0.802
	N1+N2	9 (50)	1 (14.3)		7 (43.8)	3 (33.3)		5 (33.3)	5 (50)		9 (40.9)	1 (33.3)	
**Pathological disease stage**	0+I+II	9 (50)	6 (85.7)	0.102	9 (56.2)	6 (66.7)	0.610	10 (66.7)	5 (50)	0.405	13 (59.1)	2 (66.7)	0.802
	III	9 (50)	1 (14.3)		7 (43.8)	3 (33.3)		5 (33.3)	5 (50)		9 (40.9)	1 (33.3)	
**Response to therapy (Mandrad)**	responders (TRG1+TRG2)	2 (11.1)	1 (14.3)	0.826	2 (12.5)	1 (11.1)	0.918	2 (13.3)	1 (10)	0.802	3 (13.6)	0 (0)	0.495
	non-responders (TRG3+TRG4)	16 (88.9)	6 (85.7)		14 (87.5)	8 (88.9)		13 (86.7)	9 (90)		19 (86.4)	3 (100)	

n – total number of LARC patients
nCRT – neoadjuvant chemoradiotherapy
TRG – tumor regression grade
$p<0.05$ is shown in bold

Low and high level of relative expression refer to higher or lower that median (H19: median – 0.014, NEAT1: median - 0.331, MALAT: median — 15.261, HOTAIR: median – 0.0005). Median values are reported as 2^{-dCt}.

Supplement Table S3. Mean relative expression of H19, NEAT1, MALAT1 and HOTAIR in tumor and non-tumor tissue of LARC patients before and after nCRT

lncRNA	Before nCRT (mean expression ± SD) median		Ratio of mean expression (tumor / non-tumor)	After nCRT (mean expression ± SD) median		Ratio of mean expression (tumor / non-tumor)
	tumor	non-tumor		tumor	non-tumor	
**H19**	0.054 ± 0.121<br>0.004	0.003 ± 0.004<br>0.001	18	0.150 ± 0.321<br>0.014	0.251 ± 1.166<br>0.005	0.597
**NEAT1**	0.251 ± 0.272<br>0.190	0.237 ± 0.130<br>0.249	1.059	0.448 ± 0.384<br>0.331	0.326 ± 0.193<br>0.324	1.374
**MALAT1**	11.116 ± 9.320<br>7.383	8.961 ± 5.381<br>8.396	1.240	18.039 ± 12.228<br>15.261	14.091 ± 9.501<br>13.814	1.280
**HOTAIR**	0.0004 ± 0.0005<br>0.0002	0.004 ± 0.107<br>0.0004	0.100	0.003 ± 0.009<br>0.0005	0.0002 ± 0.0002<br>0.0002	15

Values are presented as 2^{-dCt}

Supplement Table S4. Impact of H19, NEAT1, MALAT1 and HOTAIR expression before and after nCRT on overall and disease free survival outcomes in LARC patients

		Overall survival		Disease free survival	
	**Variable**	**HR (95% CI)**	***p***	**HR (95% CI)**	***p***
**Tumor before nCRT**	**H19**	0.377 (0.000-6358.265)	0.844	6.350 (0.107-378.320)	0.375
	**NEAT1**	0.830 (0.019-35.607)	0.923	1.626 (0.253-10.434)	0.608
	**MALAT1**	0.997 (0.897-1.109)	0.959	0.992 (0.923-1.066)	0.823
	**HOTAIR**	9164566829.482 (0-)	0.982	0.000 (0-)	0.821
**Tumor after nCRT**	**H19**	0.383 (0.004-35.133)	0.677	3.103 (0.493-19.519)	0.228
	**NEAT1**	12.137 (1.041-141.531)	**0.046**	5.481 (0.517-58.058)	0.158
	**MALAT1**	1.076 (0.999-1.158)	0.053	1.020 (0.957-1.088)	0.539
	**HOTAIR**	11291,052080 (0-2.867E+039)	0.822	17755158177.863 (0.000-3.720E+34)	0.409

nCRT – neoadjuvant chemoradiotherapy
HR (95% CI) – Hazard Ratio with 95% Confidence Interval

Supplement Table S5. Raw cycle threshold values of the endogenous control GAPDH in analyzed samples

Sample ID	GAPDH Ct values			
	tumor before nCRT	non-tumor before nCRT	tumor after nCRT	non-tumor before nCRT
1	20,315	21,990	23,499	20,892
2	21,724	24,424	22,409	24,199
3	21,607	23,697	25,579	22,928
4	22,097	22,873	21,546	23,108
5	20,382	28,532	22,576	23,394
6	21,944	22,974	23,376	23,111
7	21,342	22,804	21,888	23,989
8	21,952	23,022	23,964	23,552
9	22,374	24,213	21,872	25,076
10	22,543	22,762	22,492	24,471
11	22,035	23,597	21,980	22,594
12	20,855	25,685	23,126	24,030
13	24,028	23,395	21,911	24,435
14	22,707	24,634	24,515	24,791
15	22,538	22,992	23,085	23,192
16	22,789	25,805	25,356	21,031
17	23,964	22,897	23,275	22,759
18	23,083	24,061	20,640	22,837
29	23,294	23,414	24,082	24,325
20	23,137	23,114	23,403	22,824
21	21,681	22,318	24,935	22,009
22	20,931	21,594	24,264	21,743
23	26,442	28,169	20,666	20,374
24	19,656	22,118	20,987	20,644
25	20,994	19,897	22,283	22,272

Ct - Cycle threshold

Supplement Table S6. Details of publicly accessible datasets obtained from the Gene Expression Omnibus (GEO) database at the National Center for Biotechnology Information (NCBI)

Dataset	Number of samples	Number of responders	Number of non-responders	Method used	Reference	Comment
**GSE145666**	6	3	3	Agilent-067406 Human CBC lncRNA + mRNA microarray V4.0	-	
**GSE150082**	39	15	24	Agilent-026652 Whole Human Genome Microarray 4x44K v2	Sendoya JM, Iseas S, Coraglio M, Golubicki M et al. Pre-Existing Tumoral B Cell Infiltration and Impaired Genome Maintenance Correlate with Response to Chemoradiotherapy in Locally Advanced Rectal Cancer. *Cancers (Basel)* 2020;12(8).	
**GSE116742**	30	18	12	Agilent-039494 SurePrint G3 Human GE v2 8x60K Microarray	Ferrando L, Cirmena G, Garuti A, Scabini S, Grillo F, Mastracci L, Isnaldi E, Marrone C, Gonella R, Murialdo R, Fiocca R, Romairone E, Ballestrero A, Zoppoli G. Development of a long non-coding RNA signature for prediction of response to neoadjuvant chemoradiotherapy in locally advanced rectal adenocarcinoma. PLoS One 2020;15(2):e0226595.	Dworak tumor regression grade was used
**GSE145037**	31	20	11	Affymetrix Human Gene Expression Array	Zhang Y, Gao Q, Wu Y, Peng Y et al. Hypermethylation and Downregulation of UTP6 Are Associated With Stemness Properties, Chemoradiotherapy Resistance, and Prognosis in Rectal Cancer: A Co-expression Network Analysis. *Front Cell Dev Biol* 2021;9:607782.	

Supplement Table S7. Details of the web-based platforms used for analysis of the expression of the lncRNAs of interest in rectal adenocarcinoma and normal tissue, prognostic and predictive potential in LARC.

Platform	Database	Link	Reference	Note
**UCSC Xena**	TCGA	https://xenabrowser.net/	Goldman MJ, Craft B, Hastie M, Repečka K, McDade F, Kamath A, Banerjee A, Luo Y, Rogers D, Brooks AN, Zhu J, Haussler D. Visualizing and interpreting cancer genomics data via the Xena platform. Nat Biotechnol. 2020;38(6):675-678.	It is possible to restrict analysis to stage II and III of rectum adenocarcinoma patients
**TNMplot**	TCGA, GTEx, TARGET, GEO	https://tnmplot.com/analysis/	Bartha Á, Győrffy B. TNMplot.com: A Web Tool for the Comparison of Gene Expression in Normal, Tumor and Metastatic Tissues. Int J Mol Sci. 2021;22(5):2622.	
**GEPIA2**	TCGA, GTEx	http://gepia2.cancer-pku.cn/#index	Tang Z, Li C, Kang B, Gao G, Li C, Zhang Z. GEPIA: a web server for cancer and normal gene expression profiling and interactive analyses. Nucleic Acids Res. 2017;45(W1):W98-W102.	
**KMplotter**		https://kmplot.com/analysis/	Győrffy B. Integrated analysis of public datasets for the discovery and validation of survival-associated genes in solid tumors. Innovation (Camb). 2024;5(3):100625.<br><br>Lánczky A, Győrffy B. Web-Based Survival Analysis Tool Tailored for Medical Research (KMplot): Development and Implementation. J Med Internet Res. 2021;23(7):e27633.	
**ROC Plotter**		https://rocplot.com/	Tibor Fekete J, Győrffy B. A unified platform enabling biomarker ranking and	

			validation for 1562 drugs using transcriptomic data of 1250 cancer cell lines. Comput Struct Biotechnol J. 2022;20:2885-2894.	

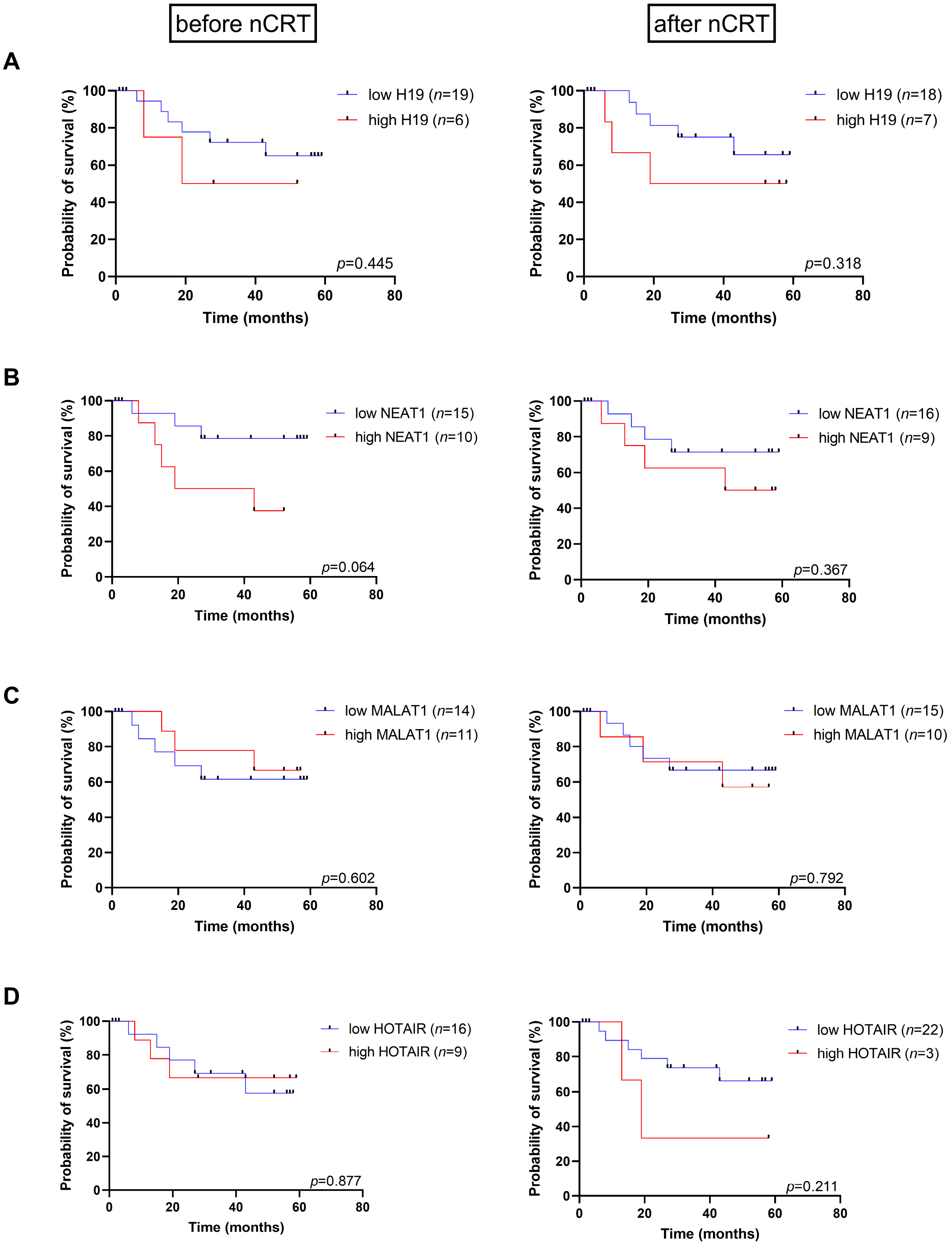

Supplement Figure S1 Kaplan-Meier curves of disease free survival in patients with locally advanced rectal cancer depending on H19 (A), NEAT1 (B), MALAT1 (C) and HOTAIR (D) expression levels before and after neoadjuvant chemoradiotherapy (nCRT). Low and high level of relative expression refer to higher or lower than median

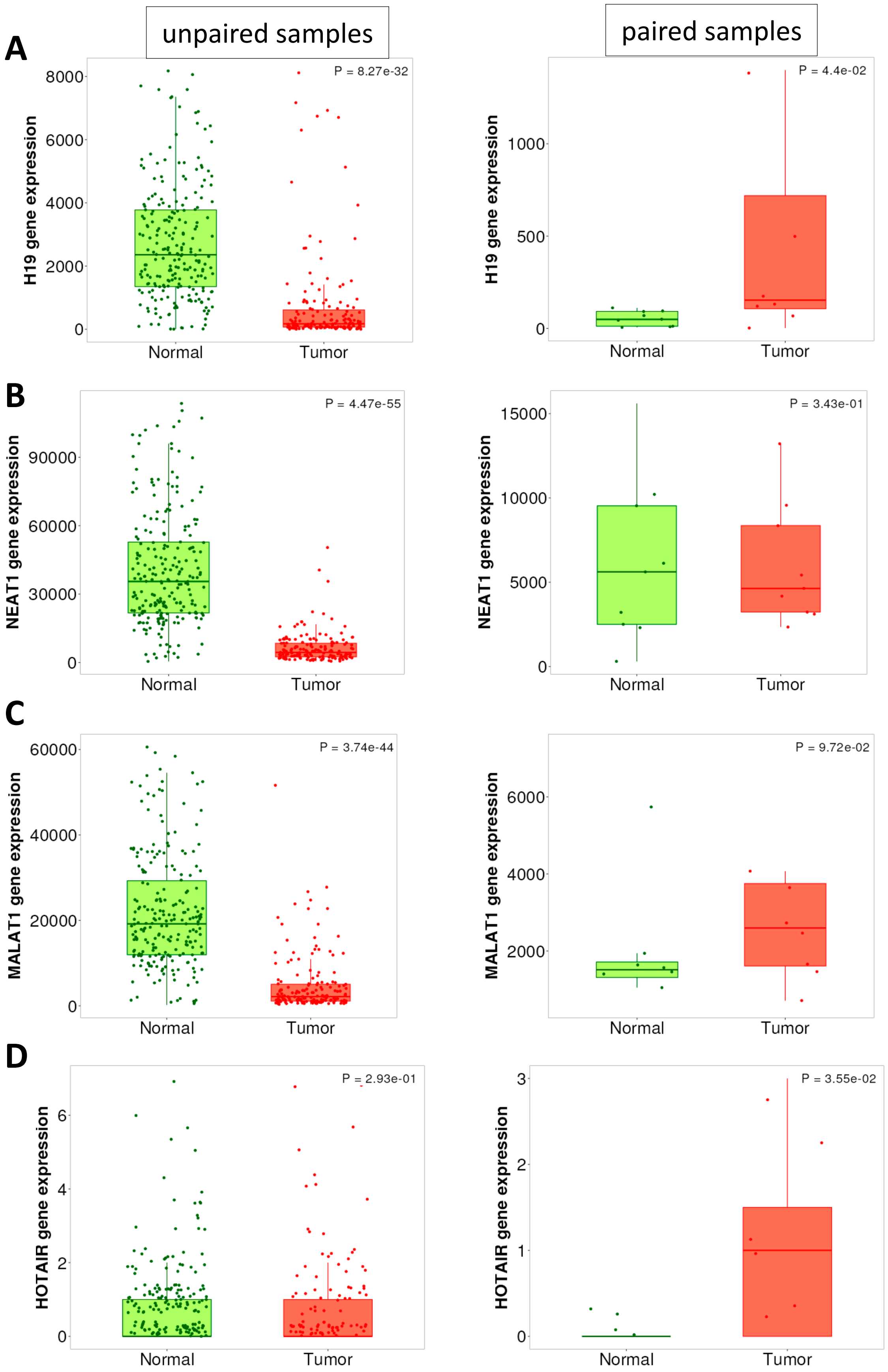

Supplement Figure S2 Expression levels of lncRNAs H19 (A), NEAT1 (B), MALAT1 (C) and HOTAIR (D) in unpaired and paired tumor and non-tumor tissue of patients with rectal cancer, analyzed by TNMplot.

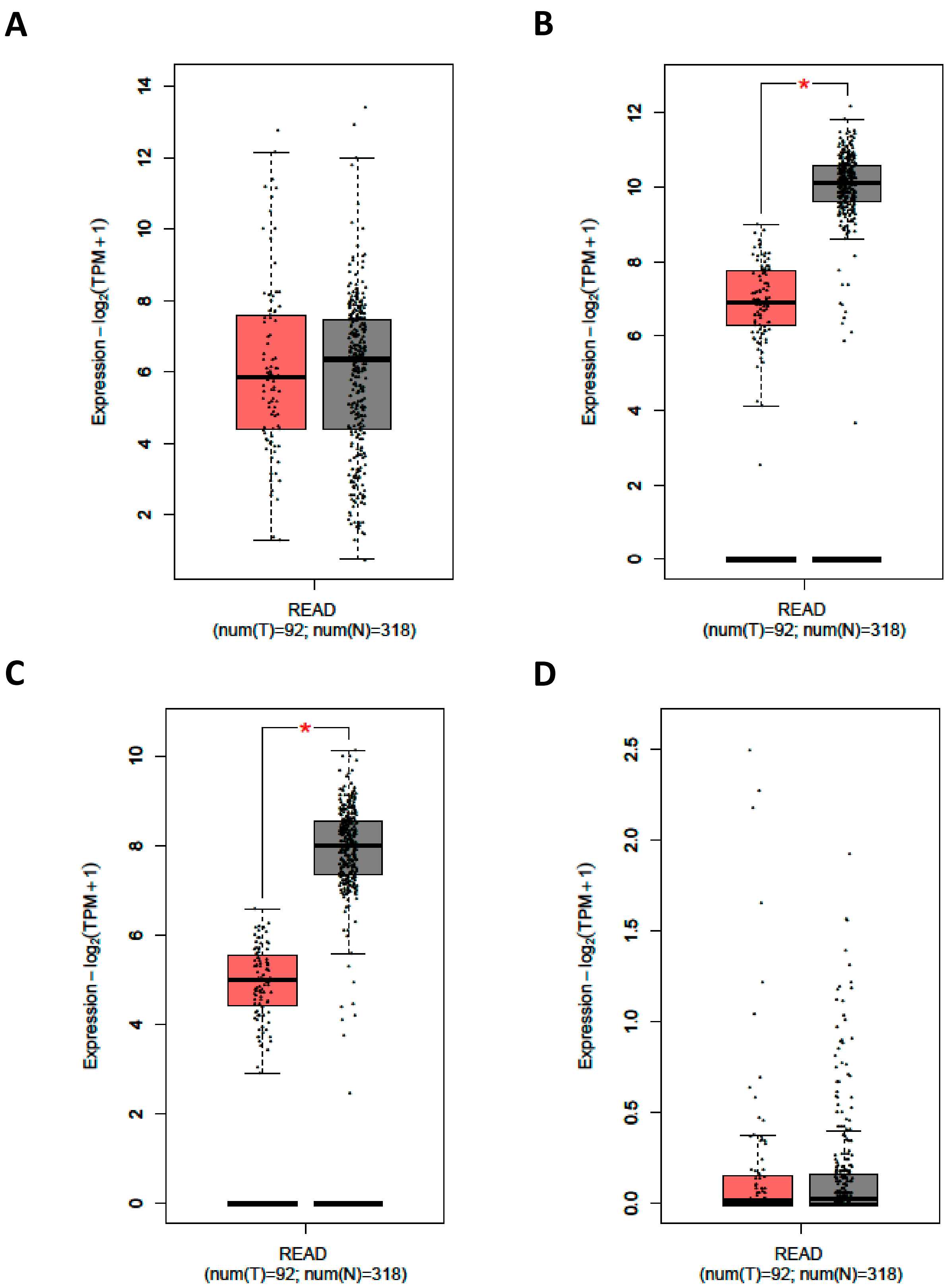

Supplement Figure S3 Expression levels of lncRNAs H19 (A), NEAT1 (B), MALAT1 (C) and HOTAIR (D) in tumor and non-tumor tissue of patients with rectal cancer, analyzed by GEPIA2. TCGA and GTEx data were used for the analysis.

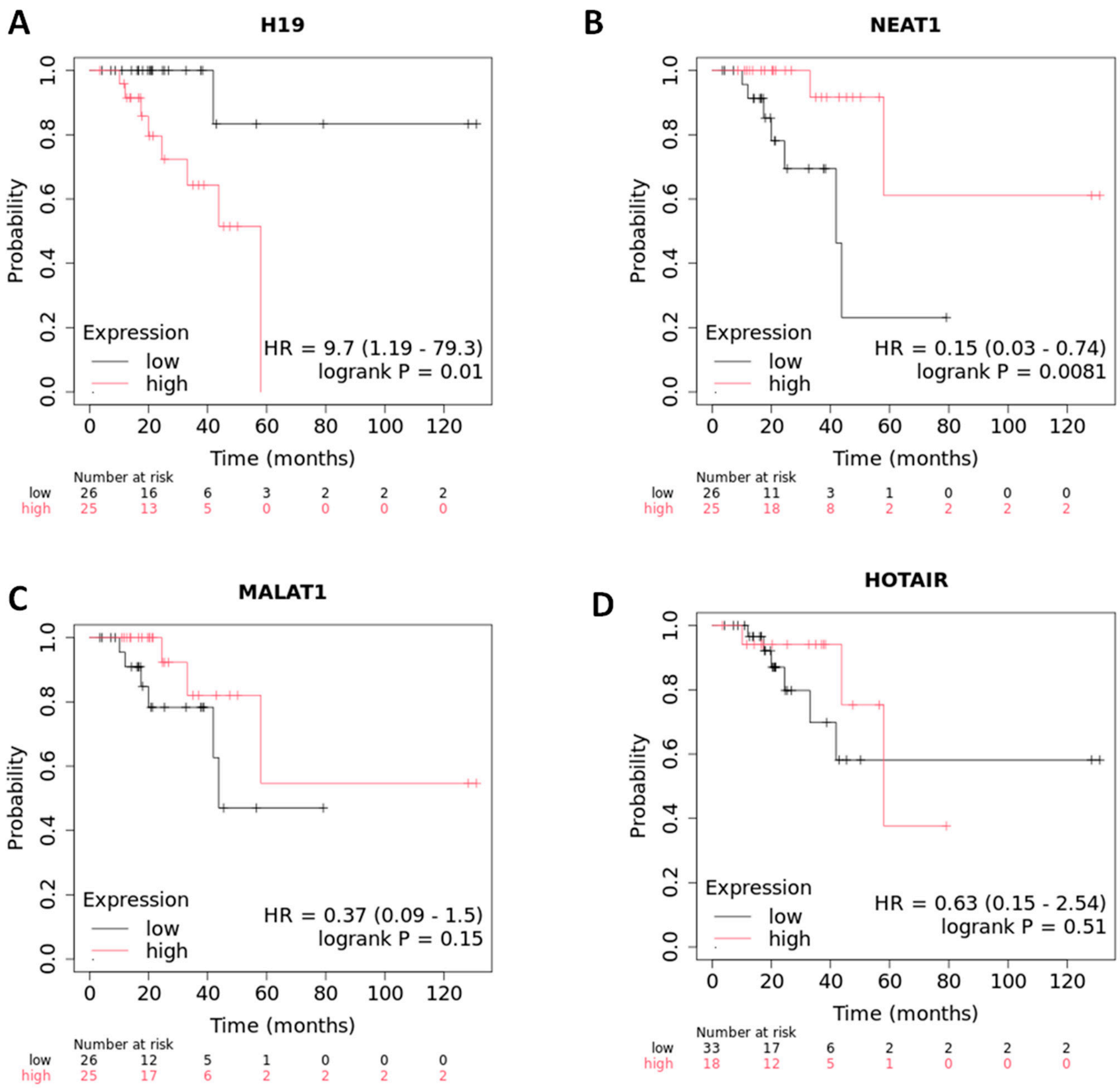

Supplement Figure S4 Kaplan-Meier curves of overall survival in patients with locally advanced rectal cancer depending on H19 (A), NEAT1 (B), MALAT1 (C) and HOTAIR (D) expression levels, analyzed by KMplot.
Low and high level of relative expression refer to higher or lower that median.

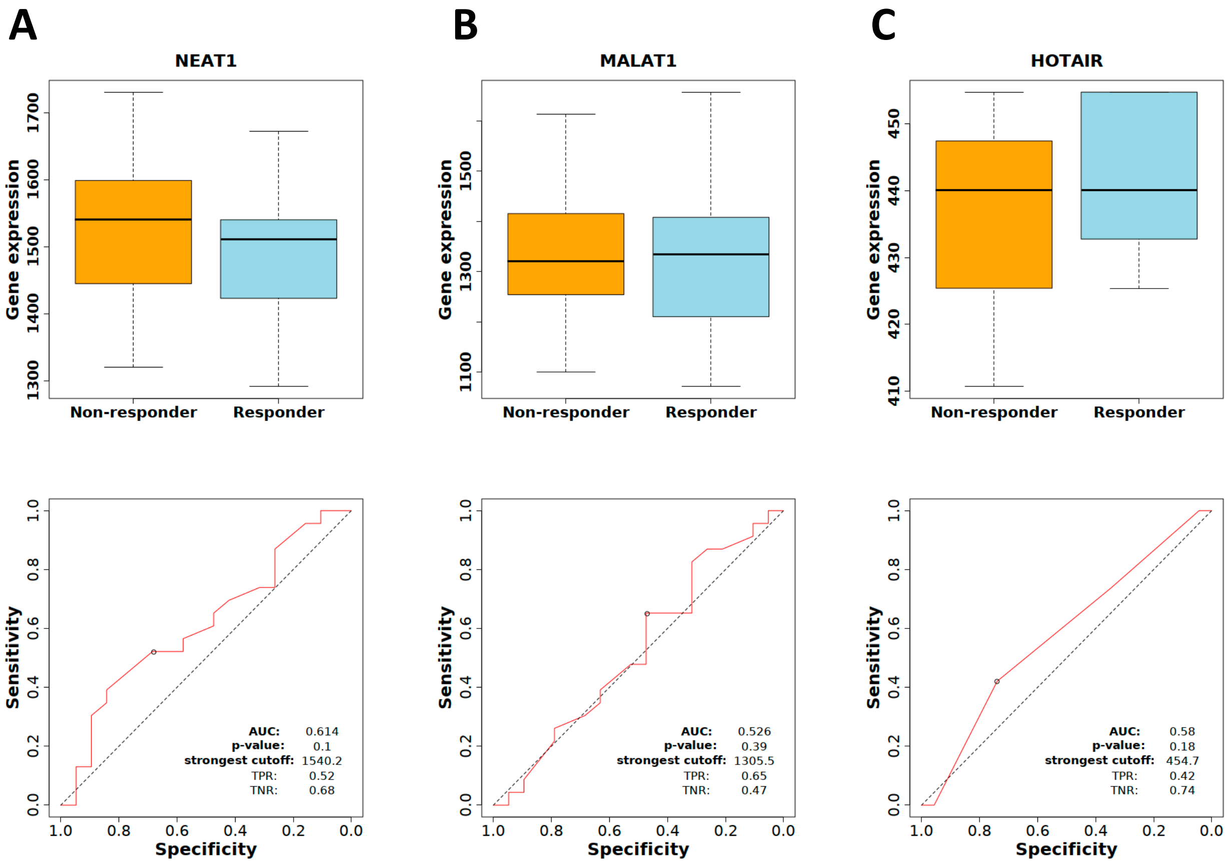

Supplement Figure S5 Predictive potential of NEAT1 (A), MALAT1 (B) and HOTAIR (C) expression in rectal adenocarcinoma patients underwent chemoradiotherapy, analyzed by ROC plotter. Data for H19 were not available on the day of access to ROC Plotter.
